# Large Language Model-Assisted Multimodal Federated Sensing for Compliance Risk Screening in Cross-Border Trade

**DOI:** 10.3390/s26216806

**Published:** 2026-10-28

**Authors:** Xinyi He, Yifan Yang, Yuening Wang, Qingxi Meng, Yanping Zhang, Jingke Liang, Manzhou Li

**Affiliations:** 1 China Agricultural University, Beijing 100083, China; 2 Peking University, Beijing 100871, China; 3 Desert Comprehensive Management Center of Bayannur City, Bayannur 015099, China

**Keywords:** heterogeneous multimodal sensing, federated learning, regulation-aware risk assessment, LLMs, agricultural product safety

## Abstract

With the continuous expansion of cross-border agricultural supply chains, reliable food-safety risk screening under decentralized data ownership, heterogeneous sensing equipment, and jurisdiction-dependent, non-stationary regulatory limits pose a fundamental challenge to intelligent inspection systems. To address these limitations, this paper proposes an LLM-assisted regulation-aware heterogeneous multimodal federated learning framework that establishes a three-tier decoupling architecture. First, an available-modality and quality-adaptive fusion mechanism dynamically weights RGB images, near-infrared (NIR) spectra, and environmental time-series while enforcing strict zero weighting on unavailable modalities and continuous suppression of degraded observations. Second, a regulation-independent state-prototype collaboration and personalized federated learning strategy is developed on a unified continuous concentration scale, enabling cross-institutional transfer of physical contamination representations while avoiding the use of jurisdiction-dependent compliance labels to define shared prototype groups and preserving client-specific adapters against non-IID drift. Third, offline large language model (LLM) parsing transforms unstructured multi-jurisdictional trade regulations into machine-executable rule bases, which are coupled with an uncertainty-aware risk calibration module to dynamically determine exceedance probabilities and reinspection recommendations without retraining front-end sensing encoders when regulatory standards shift. Experimental evaluations on a multisource maize and aflatoxin B1 screening dataset in a workstation-based federated simulation demonstrate that the proposed framework achieves a mean absolute error (MAE) of 0.054±0.002 and a root mean square error (RMSE) of 0.078±0.003, while elevating the area under the curve (AUC) and F1-score to 0.862±0.008 and 0.833±0.009, respectively, reducing the false negative rate (FNR) to 0.121±0.007 (p<0.001). Under severe client heterogeneity and missing-modality scenarios, the proposed approach maintains an AUC of 0.823±0.010 and an F1-score of 0.796±0.011. Ablation and ownership-level analyses assess robustness, calibration (expected calibration error of 0.032), and regulatory adaptation with fixed sensing encoders, providing an end-to-end, traceable decision-support paradigm for international agricultural trade screening.

## 1. Introduction

Agricultural products pass through producers, storage facilities, processors, and inspection agencies before reaching export or import markets. Mycotoxin contamination can affect consumer health and lead to shipment rejection and economic losses [1]. Compliance depends on both contaminant concentration and the applicable commodity and intended-use category: Commission Regulation (EU) 2023/915, for example, specifies different aflatoxin limits for different cereal products and uses [2]. Laboratory analysis remains the reference for quantification, while NIR-based models have been investigated for maize screening, including aflatoxin B1 estimation [3] and fumonisin regression followed by comparison with a legal limit [4]. These studies motivate rapid screening but do not establish performance across arbitrary sensors or commodities. In distributed agricultural settings, data ownership, heterogeneous observations, communication constraints, and limited computing resources also affect collaborative learning [5,6]. This study considers how multimodal sensing, federated collaboration, and destination-specific rules can be combined for maize and aflatoxin B1 screening.

Three design issues motivate the framework. First, a fixed-concatenation implementation does not explicitly distinguish an absent sensor from a present but degraded observation. Missing-modality learning is already studied in multimodal FL [7,8]; our focus is to combine explicit availability masking with quality-dependent weighting for the sensing modalities used here. Second, FedAvg aggregates model parameters [9], while FedProx adds a proximal term to address heterogeneous local optimization [10]. Neither operation by itself defines how jurisdiction-specific compliance labels should be reconciled. If the same concentration is labeled differently under two applicable limits, directly aggregating compliance-class prototypes can mix different meanings. We therefore organize collaboration by common concentration intervals and retain local adapters. Third, a classifier trained for one legal threshold does not explicitly expose the concentration estimate needed to apply another threshold. A concentration regressor followed by a replaceable threshold already separates these operations [4]; our additional design question is how to condition screening risk on predictive dispersion and structured regulatory context. The gap addressed here is this joint screening design, rather than the absence of missing-modality, personalization, or threshold-based methods in prior work.

To address these design issues, this paper proposes an LLM-assisted regulation-aware heterogeneous multimodal federated learning framework based on a three-tier decoupling architecture. The framework establishes an integrated hierarchy that decouples physical multimodal feature fusion, cross-institutional state collaboration, and regulation-conditioned compliance calibration. First, an available-modality and quality-adaptive fusion module dynamically maps available nonempty subsets of RGB images, NIR spectra, and environmental time-series into a unified contamination-state space, enforcing strict zero weighting on missing modalities while continuously attenuating low-quality observations. Second, a regulation-independent state-prototype collaboration mechanism aggregates physical contamination states on a unified continuous concentration scale rather than conflicting binary compliance labels, while client-specific personalized adapters mitigate non-IID data drift. Third, an offline LLM-assisted regulatory parsing pipeline extracts structured, executable compliance rules from complex trade texts, enabling an uncertainty-aware risk calibration network to dynamically evaluate exceedance probabilities and generate reinspection recommendations without retraining front-end encoders when regulations shift. The primary contributions of this paper are summarized as follows:

1. Available-Modality and Quality-Adaptive Multimodal Fusion: We design a unified latent mapping architecture equipped with an availability-gated mechanism and a feature-statistic quality estimation network. The module dynamically penalizes degraded observations and enforces strict zero weighting on unavailable modalities, supporting the modality-availability configurations evaluated across distributed supply chain nodes.
2. Regulation-Independent Prototype Collaboration and Personalized FL: We develop a prototype collaboration mechanism that shares contamination representations indexed by concentration over a continuous concentration scale, separating physical contamination estimation from jurisdiction-dependent regulatory semantics. By combining support-weighted prototype aggregation with local bottleneck adapters, the framework avoids grouping prototypes by local compliance labels and supports adaptation to non-IID client data.
3. LLM-Assisted Parsing and Regulation-Conditioned Risk Calibration: We establish an offline regulatory knowledge extraction workflow utilizing large language models with schema-constrained verification, transforming unstructured international trade standards into machine-executable conditions. A regulation-conditioned calibration module integrates continuous concentration predictions, predictive dispersion, and validated regulatory limits to generate calibrated exceedance risks and confidence-bounded reinspection recommendations, accommodating regulatory updates without retraining the sensing network.

## 2. Related Work

### 2.1. Multimodal Sensing and Agricultural Contaminant Detection

NIR-based maize studies provide task-specific precedents for contamination screening: Wang et al. [3] estimate aflatoxin B1 using spectral representations and convolutional networks, whereas Della Riccia and Del Zotto [4] use multivariate regression to estimate fumonisins and assess samples against a legal limit. These results concern particular sensing and contaminant settings. Multimodal representation learning provides a broader basis for combining complementary inputs [11], but it should not be characterized as universally requiring complete observations. FedMultimodal explicitly benchmarks multimodal FL under missing modalities, missing labels, and erroneous labels [7]. Cao et al. [8] address missing modalities in heterogeneous multimodal FL through mask restoration and self-guidance. Thus, handling missing inputs is established prior work. Our fusion design combines a strict availability mask with learned quality-dependent weights for RGB, NIR, and environmental observations, with laboratory referral when no modality is available. Its contribution is this task-specific integration, not the introduction of modality masking itself.

### 2.2. Heterogeneous and Personalized Federated Learning

Agricultural FL reviews identify data heterogeneity, communication constraints, and data governance as deployment challenges [5,6]. Arbaoui et al. [12] organize FL research around personalization, optimization, robustness, and aggregation strategies. Caruccio et al. [13] provide a complementary multi-criteria categorization of FL approaches. These surveys guide the distinction between established method families; the individual mechanisms are attributed to their primary sources. FedAvg exchanges and averages model parameters [9]; FedProx adds proximal regularization to local optimization [10]; Ditto learns personalized models with regularization toward a global model [14]; and FedProto exchanges class prototypes and constrains local representations using aggregated prototypes [15].

Prototype exchange and personalization are therefore not new in themselves. The extension considered here is to index prototypes by shared physical concentration intervals instead of jurisdiction-dependent compliance classes, and to combine these prototypes with client-specific adapters and observation-quality-dependent aggregation weights. For a fixed measured batch, changing the applicable legal limit changes the compliance decision but not its measured concentration. Using the latter to define the prototype groups avoids assigning the groups by conflicting local compliance labels. This construction still requires common units and interval definitions; it does not imply that all statistical heterogeneity or representation mismatch is eliminated. Table 1 summarizes the mechanisms inherited from prior work and the scope of the present integration.

### 2.3. Regulatory Knowledge-Driven and Uncertainty-Aware Decision-Making

Regulatory knowledge extraction and probabilistic calibration address different parts of the decision process. Li and Maiti [16] study LLM analysis combined with backend web services for continuous compliance checks, including rule identification and numeric checking. Their work supports the use of LLM-assisted regulatory processing, and it does not claim that every regulatory change requires model retraining. In this study, regulatory text is processed offline into validated structured rules; online screening retrieves the applicable rule and combines it with the predicted contamination state. The commodity and use restrictions in the rule must remain traceable to the source regulation [2].

Raza et al. [17] use case-based reasoning for database workload performance prediction and adaptation. The limited conceptual connection is the use of predicted system behavior to inform a context-dependent operational response. Their study concerns database workloads, does not evaluate agricultural contamination or federated sensing, and is not evidence for the accuracy of our screening model. Our method does not implement their case-retrieval algorithm: it applies an explicit regulatory condition to the estimated contamination state.

**Table 1:** Mechanism-level comparison with related work. The table distinguishes established components from their integration in this study; it is not a ranking of empirical performance. Bold identifies the row describing this study.

Prior Work	Established Mechanism	Scope in the Cited Work	Extension in This Study
FedMultimodal [7]; Cao et al. [8]	Missing-modality benchmarking; mask restoration and self-guidance	Multimodal FL with incomplete observations	Joint availability masking and quality weighting for RGB, NIR, and environmental sensing
FedAvg [9]; FedProx [10]	Parameter averaging; proximal local objective	Distributed optimization with heterogeneous local data	Concentration-indexed prototype exchange rather than encoder-parameter exchange
Ditto [14]	Regularization-based personalized models	Client-specific learning around a global model	Local adapters combined with a shared contamination-state prototype bank
FedProto [15]	Aggregated class prototypes and local prototype regularization	Collaboration through class-indexed representations	Common concentration intervals and support/quality weighting, with legal decisions applied downstream
Li and Maiti [16]	LLM-assisted rule processing with backend services	Continuous compliance checking	Offline validated rule compilation and online rule retrieval coupled to sensing estimates
Guo et al. [18]	Post hoc confidence calibration, including temperature scaling	Classifier confidence adjustment	Risk conditioning on regulatory margin, dispersion, and structured rule context
**This study**	**Integration of the above mechanism families**	**Maize/AFB1 screening in a simulated federated setting**	**Separates sensing, concentration-state collaboration, and regulation-conditioned decisions**

Post hoc calibration is also established. Guo et al. [18] investigate neural-network confidence calibration, including temperature scaling. A scalar temperature adjusts classifier logits but does not itself encode a commodity-specific legal limit. Our conditional risk module instead takes the regulatory margin, predictive dispersion, and structured rule context as inputs. This is a different conditioning design, not a guarantee that its outputs are calibrated; calibration quality is assessed in Section 4.6. Together, availability- and quality-aware fusion, concentration-indexed prototype exchange with local adaptation, and offline rule-conditioned risk estimation define the scope of this paper’s contribution.

## 3. Materials and Method

### 3.1. Data Collection

The dataset employed in this study consists of publicly available NIR spectral benchmarks, self-collected multimodal agricultural sensing observations, gold-standard laboratory chemical measurements, and statutory regulatory corpora, with cross-border maize batches serving as the primary investigation object, as summarized in Table 2. To faithfully reflect distributed ownership across realistic supply chains, self-collected data were acquired across three geographically distinct facilities: the Changping Agricultural Experimental Base in Beijing (production stage), a commercial grain storage and milling complex in Baoding, Hebei Province (storage and primary processing stage), and an import–export agricultural testing center in Weifang, Shandong Province (pre-customs inspection stage). Across these three institutional owners, ten operational federated clients were established based on physical asset boundaries and independent sensing infrastructures: Changping comprises three farm-level cooperative silos (Clients 1–3, contributing 420 batches); Baoding comprises four independent storage silos equipped with automated environmental monitoring nodes (Clients 4–7, contributing 560 batches); and Weifang comprises three parallel analytical screening lines (Clients 8–10, contributing 420 batches). In total, 1400 independent physical maize batches were tracked and sampled continuously from September 2025 to February 2026, spanning intake, long-term storage, and outbound dispatch.

For each of the 1400 independent batches, multi-point cross-sectional sampling was executed to address intra-batch spatial heterogeneity of mycotoxins. Specifically, nine standardized high-resolution RGB images were captured per batch under uniform diffuse LED illumination (yielding 12,600 images total) using a Basler acA2440-35uc industrial camera (Basler AG, Ahrensburg, Germany; 2448×2048 resolution) to characterize pericarp morphology, mold discoloration, and kernel damage. Simultaneously, six repeated near-infrared diffuse reflectance spectra were acquired per batch across the 900–1700 nm range using a Specim FX17 imaging spectrometer (Specim, Spectral Imaging Ltd., Oulu, Finland), and they were subsequently averaged to suppress localized kernel placement noise (yielding 8400 spectra total). Ambient warehouse microclimates were tracked continuously via Sensirion SHT35 sensor nodes (Sensirion AG, Staefa, Switzerland) deployed in storage units logging at 10-min intervals from 1 September 2025, 00:00 to 1 March 2026, 12:00, an elapsed duration of 181.5 days (4356 h). Two primary sensor nodes were used, and the reported totals are 52,272 records for each environmental variable, as listed in Table 2. Definitive ground-truth concentrations of aflatoxin B1 (AFB1) were quantified via high-performance liquid chromatography coupled with fluorescence detection (HPLC-FLD) following standard immunoaffinity clean-up procedures with a limit of detection (LOD) of 0.05μg/kg. Public maize NIR spectra (4800 records) were utilized strictly as an auxiliary corpus for spectral representation pretraining and were never blended into client datasets.

In parallel, a statutory regulatory corpus comprising 326 binding legal clauses was compiled from European Commission Regulation (EU) 2023/915 and Chinese National Standard GB 2761, detailing maximum levels across commodity subtypes, processing stages (e.g., direct human consumption vs. sorting and physical treatment), and effective dates. This data architecture establishes an unambiguous boundary between objective physical contamination sensing and legal compliance conditions, providing the prerequisite for regulation-independent federated collaboration.

### 3.2. Data Augmentation and Preprocessing

Because multimodal observations diverge fundamentally in dimensional scale, acquisition frequency, and signal-to-noise ratio, direct concatenation would bias representation learning toward modalities with dominant amplitudes or higher sampling rates. A standardized preprocessing pipeline is therefore applied before feature projection. For NIR spectra $x^{s}\in R^{d}$, abnormal edge bands subject to atmospheric moisture absorption are truncated, followed by mean-variance standardization based strictly on training-set statistics: ${\tilde{x}}_{j}^{s}=\left(x_{j}^{s}-\mu_{j}\right)/\left(\sigma_{j}+\epsilon \right)$, with $\epsilon =10^{-6}$ ensuring numerical stability. For RGB images, pixels $x_{h,w,c}^{v}$ are scaled to [0,1] and channel-wise normalized: ${\tilde{x}}_{h,w,c}^{v}=\left(x_{h,w,c}^{v}/255-\mu_{c}\right)/\left(\sigma_{c}+\epsilon \right)$. Environmental time-series are temporally aligned with physical batch inspection timestamps within a temporal window Δt=30 min, a parameter empirically justified by the substantial thermal inertia of bulk grain piles where ambient shifts manifest gradually over multi-hour intervals.

To prevent overfitting and simulate realistic operational perturbations, physically grounded data augmentations are introduced during training. For RGB images, random affine rotations ($\pm 15^{\circ }$), horizontal and vertical flips, and subtle brightness jitter (±10%) simulate varying optical inspection angles and lighting variations. For NIR spectra, zero-mean Gaussian perturbations are injected: ${\tilde{x}}_{\mathrm{aug}}^{s}={\tilde{x}}^{s}+\eta$, where $\eta ∼N(0,\sigma_{n}^{2}I)$ with $\sigma_{n}=0.02$, corresponding to the empirical dark noise floor (≈1.5--2.0%) of industrial InGaAs focal plane detectors. Furthermore, to explicitly train the network for intermittent sensor dropouts, modality random dropout is enforced during training: for modality *q*, an independent Bernoulli mask $z^{q}∼\mathrm{Bernoulli}\left(1-p_{q}\right)$ is applied, where the default dropout rate is set to $p_{q}=0.2$ (and varied between 0.1 and 0.5 in sensitivity analyses). Modality masks are formally denoted as $m_{i}=\left[m_{i}^{1},\ldots ,m_{i}^{M}\right]\in {\left\{0,1\right\}}^{M}$, where $m_{i}^{q}=1$ denotes availability and $m_{i}^{q}=0$ indicates missingness. Validation and test sets undergo only deterministic preprocessing without augmentation, guaranteeing unbiased evaluation.

### 3.3. Proposed Method

#### 3.3.1. Overall Framework

An LLM-assisted regulation-aware heterogeneous multimodal federated learning framework is developed for compliance risk screening in cross-border agricultural trade. The preprocessed RGB images, NIR spectra, and environmental sensing data are first fed into their corresponding modality-specific encoders to extract visual, spectral, and environmental state representations, respectively. The resulting multimodal features are subsequently delivered to the available-modality and quality-adaptive fusion module, where modality-availability masks are employed to identify missing inputs and a quality estimation network is used to evaluate the reliability of the current observation from each modality. Fusion weights are then dynamically assigned according to both modality availability and observation quality, thereby producing a unified contamination-state representation. This representation is further processed by a client-specific personalized adapter to compensate for local distribution shifts caused by differences in sensing devices, geographic environments, and data distributions, after which a contaminant prediction head estimates the contaminant concentration together with its predictive uncertainty.

Meanwhile, contamination-state prototypes are extracted from local representations according to a unified concentration scale at each client. Instead of raw data or regulation-dependent compliance labels, the resulting state prototypes and their sample-support and quality statistics are communicated for server aggregation. The server performs reliability- and support-aware aggregation by considering the effective sample support and prototype reliability of individual clients, thereby constructing a global contamination-state prototype bank. The shared state knowledge is subsequently returned to the participating clients to constrain local representation learning, whereas the personalized adapters remain exclusively local. In this manner, cross-client knowledge collaboration and client-specific adaptation are jointly achieved.

After contamination-state estimation, the framework proceeds to regulation-conditioned risk assessment. An LLM is employed to assist in parsing regulatory documents and extracting structured information, including destination, commodity category, intended use, contaminant type, permissible limit, effective date, and source clause. The extracted information is validated and converted into machine-executable regulatory conditions. Finally, the predicted contaminant concentration distribution and the applicable regulatory limit are jointly introduced into the uncertainty-aware risk calibration module, where exceedance risk is estimated and samples located near the regulatory threshold or associated with insufficient evidence are explicitly identified. The final output contains a calibrated risk score, applicable regulatory basis, and reinspection recommendation, thereby establishing an end-to-end processing pipeline from multimodal sensing and federated state collaboration to regulation-constrained risk decision-making.

#### 3.3.2. Available-Modality and Quality-Adaptive Fusion

To accommodate arbitrary sensor configurations and noisy observations across participating clients, an available-modality and quality-adaptive fusion module is designed. Let $X_{i}^{q}$ represent the input observation for the *q*-th modality (q∈{1,…,M}). Modality-specific encoders $E_{q}\left(\cdot \right)$ (e.g., Vision Transformer for RGB images, 1D-CNN for NIR spectra, and MLP for environmental time-series) map inputs into initial feature spaces, which are subsequently projected into a unified latent space via projection layers $P_{q}\left(\cdot \right)$: $u_{i}^{q}=P_{q}\left(\mathrm{pool}\left(E_{q}\left(X_{i}^{q}\right)\right)\right)\in R^{d_{f}}$, with $d_{f}=256$.

To capture the intrinsic reliability of projected features without relying on external quality ground truth, a statistical quality descriptor is extracted: $c_{i}^{q}=[\mathrm{mean}\left(u_{i}^{q}\right);\mathrm{std}\left(u_{i}^{q}\right);$ $\mathrm{norm}(u_{i}^{q})]$. A lightweight quality estimation network $Q_{q}\left(\cdot \right)$, implemented as a two-layer MLP with Sigmoid output, estimates a continuous reliability score: $r_{i}^{q}=Q_{q}\left(c_{i}^{q}\right)\in \left(0,1\right)$. The quality estimation network is trained end-to-end with the primary predictive objective; features distorted by noise or instrument artifacts exhibit abnormal dispersion, resulting in lower learned reliability scores.

To resolve the issue of non-zero weighting on missing modalities identified by reviewers, we formulate an availability-gated score $g_{i}^{q}$ that guarantees strict zero weight for any missing input:

(1) $$g_{i}^{q}=\left\{\begin{array}{cc} r_{i}^{q}, & \mathrm{if}\;m_{i}^{q}=1\\ -\infty , & \mathrm{if}\;m_{i}^{q}=0 \end{array}\right..$$

**Figure 1:**
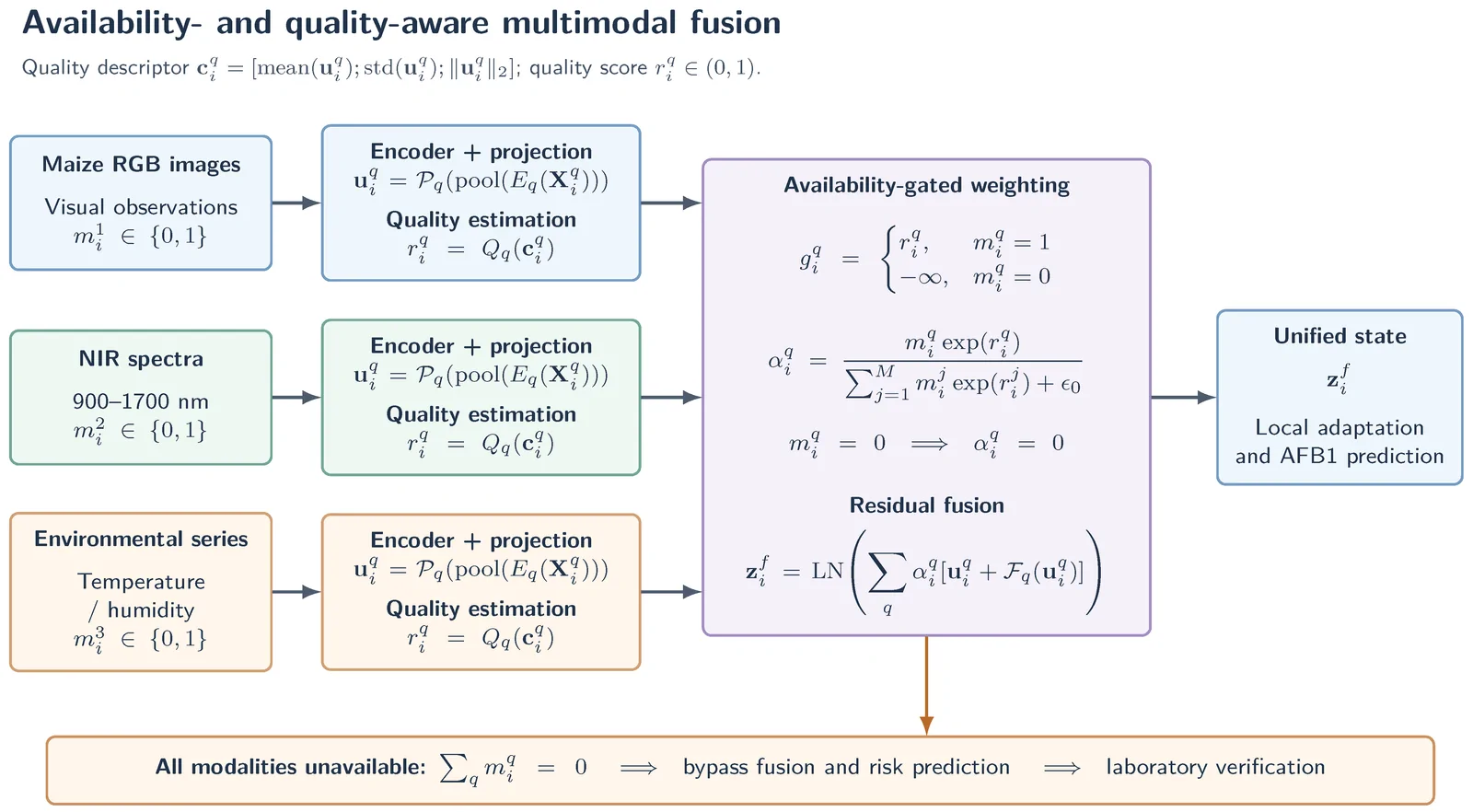
Availability- and quality-aware multimodal fusion architecture. Missing modalities are strictly masked out ($m_{i}^{q}=0\Rightarrow \alpha_{i}^{q}=0$) while available inputs are adaptively weighted based on estimated quality scores $r_{i}^{q}$. The diagram follows Equations (1)–(3); samples with no available modalities bypass prediction and are referred for laboratory verification.

In practical numerical implementation, −∞ is realized by masking missing scores with a large negative constant −K ($K=10^{4}$). The normalized adaptive fusion weight $\alpha_{i}^{q}$ across available modalities is then computed via a masked softmax:

(2) $$\alpha_{i}^{q}=\frac{m_{i}^{q}\exp\left(r_{i}^{q}\right)}{\sum_{j=1}^{M}m_{i}^{j}\exp\left(r_{i}^{j}\right)+\epsilon_{0}},$$

where $\epsilon_{0}=10^{-12}$. Under this formulation, if modality *q* is unavailable ($m_{i}^{q}=0$), its fusion weight is identically zero ($\alpha_{i}^{q}\equiv 0$). When all modalities are missing ($m_{i}^{q}=0$ for all *q*), the denominator collapses to $\epsilon_{0}$, and the system triggers an automatic “insufficient sensing data” flag, routing the sample directly to mandatory laboratory testing rather than outputting spurious risk scores. This mathematical formulation is fully consistent with the schematic gating mechanism shown in Figure 1.

The unified contamination-state representation $z_{i}^{f}\in R^{d_{f}}$ is then synthesized through residual feature transformation and layer normalization:

(3) $$z_{i}^{f}=\mathrm{LN}\left(\sum_{q=1}^{M}\alpha_{i}^{q}\left[u_{i}^{q}+F_{q}\left(u_{i}^{q}\right)\right]\right),$$

where $F_{q}\left(\cdot \right)$ denotes a modality-specific non-linear residual adapter and LN(·) enforces inter-client feature stability.

#### 3.3.3. State-Prototype Collaboration and Personalized Federated Learning

**Figure 2:**
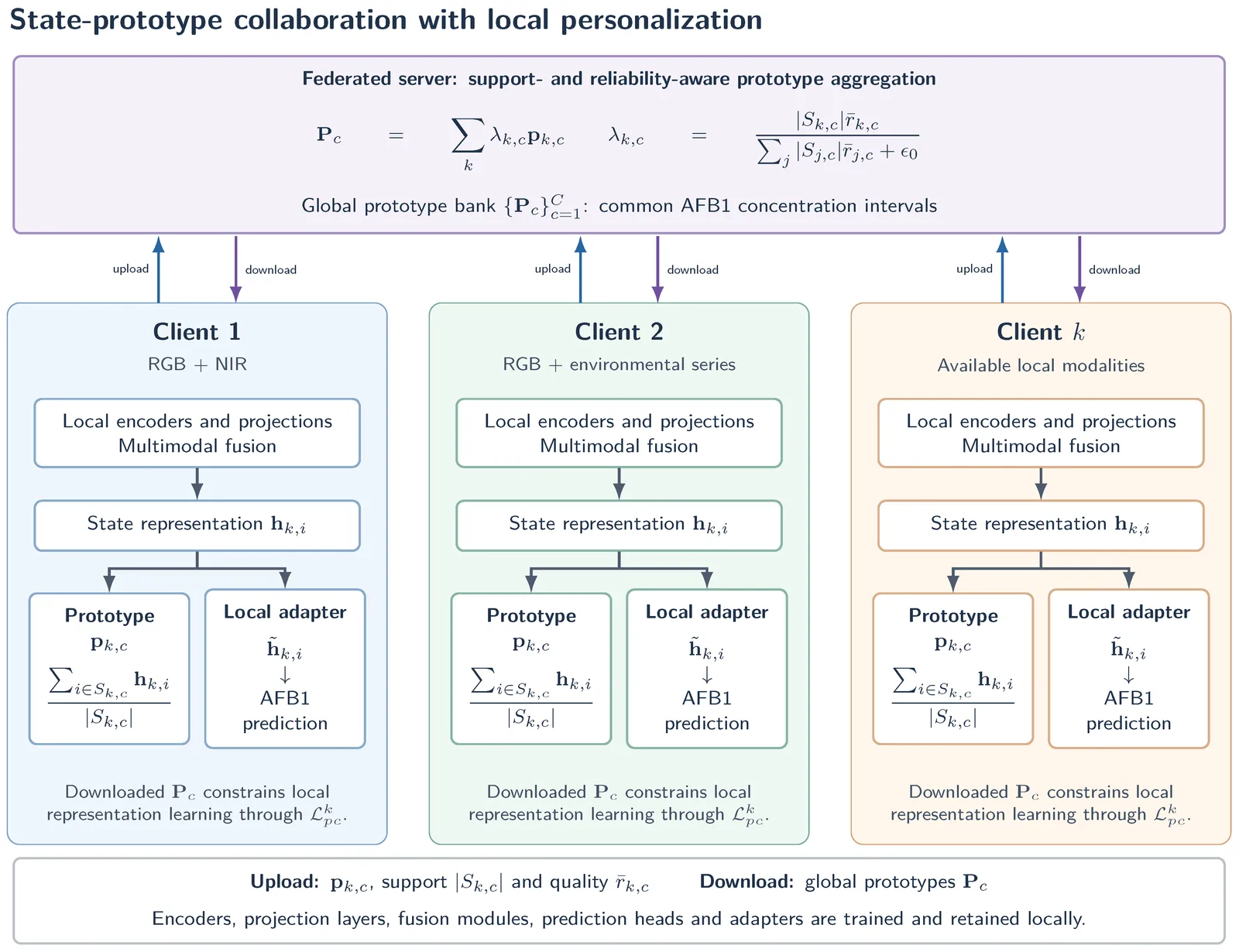
State-prototype collaboration and personalized federated learning. Clients upload state-prototype centroids $p_{k,c}$ together with sample support and observation-quality statistics, and they receive the global prototype bank $\left\{P_{c}\right\}$. Encoders, projection layers, fusion modules, prediction heads, and personalized adapters remain local. Downloaded prototypes constrain local representation learning through $L_{pc}^{k}$; no encoder weights are exchanged.

Following multimodal fusion, local representations exhibit uniform dimensionality across all clients. However, regional production environments, varying moisture baselines, and disparate storage periods introduce significant client drift. Furthermore, cross-border compliance standards define conflicting legal thresholds over identical physical contamination states. To overcome these challenges, we design a regulation-independent state-prototype collaboration protocol that decouples physical contamination knowledge from jurisdiction-dependent compliance semantics, as illustrated in Figure 2.

To resolve previous ambiguities regarding the federated communication protocol, we explicitly state that the proposed framework operates under a strict prototype-only exchange protocol. Specifically, client encoders, projection heads, and personalized adapters are updated entirely on local hardware and are never transmitted to the server. Low-dimensional contamination-state prototype vectors, sample support counts, and mean observation-quality statistics are communicated for aggregation during communication rounds, reducing the size of the transmitted representation and mitigating parameter privacy leakage.

At each local client *k*, the fused representation $z_{k,i}^{f}$ is projected into a shared contamination-state representation $h_{k,i}\in R^{d_{f}}$. To preserve client-specific characteristics against non-IID drift, a lightweight local bottleneck adapter is integrated:

(4) $${\tilde{h}}_{k,i}=h_{k,i}+W_{k}^{u}\phi \left(W_{k}^{d}h_{k,i}+b_{k}^{d}\right),$$

where $W_{k}^{d}\in R^{D_{a}\times d_{f}}$ and $W_{k}^{u}\in R^{d_{f}\times D_{a}}$ are personalized adapter matrices ($D_{a}=64$) maintained exclusively on client *k*. The adapted feature ${\tilde{h}}_{k,i}$ feeds into the local concentration regression head, whereas the shared representation $h_{k,i}$ is employed to construct state prototypes.

To avoid semantic collisions, prototypes are organized over a continuous, regulation-independent contamination scale partitioned into C=10 uniform intervals spanning 0–50μg/kg ($\left[0,c_{1}\right),\ldots ,\left[c_{C-1},c_{C}\right]$). For concentration interval *c*, the local state prototype at client *k* is calculated as the mean representation of local samples residing in that interval:

(5) $$p_{k,c}=\frac{1}{|S_{k,c}|}\sum_{i\in S_{k,c}}h_{k,i},$$

where $S_{k,c}$ denotes the local sample index set with ground-truth concentration falling in interval *c*, and $N_{min}=10$ is the minimum sample threshold required for client *k* to construct and transmit a valid prototype for state *c*. Let $S_{t}\subseteq \left\{1,\ldots ,K\right\}$ denote the randomly sampled subset of clients participating in communication round *t* ($|S_{t}|=\left\lceil qK\right\rceil$), and let $K_{c}^{\left(t\right)}=\{k\in S_{t}||S_{k,c}\left|\geq N_{min}\right\}$ represent the subset of active clients possessing sufficient sample support in state *c*. When $K_{c}^{\left(t\right)}\neq \emptyset$, the server computes a round-level aggregated prototype vector using dual support- and reliability-aware weighting:

(6) $${\tilde{P}}_{c}^{\left(t\right)}=\sum_{k\in K_{c}^{\left(t\right)}}\lambda_{k,c}^{\left(t\right)}p_{k,c}^{\left(t\right)},\;\lambda_{k,c}^{\left(t\right)}=\frac{|S_{k,c}|{\bar{r}}_{k,c}}{\sum_{j\in K_{c}^{\left(t\right)}}\left|S_{j,c}\right|{\bar{r}}_{j,c}+\epsilon_{0}},$$

where $|S_{k,c}|$ is the sample support count, ${\bar{r}}_{k,c}=\frac{1}{|S_{k,c}|}\sum_{i\in S_{k,c}}\frac{1}{M}\sum_{q=1}^{M}r_{i}^{q}$ represents the mean observation quality of client *k* in state *c*, and $\epsilon_{0}=10^{-6}$ guarantees numerical stability. The normalized weights satisfy $\sum_{k\in K_{c}^{\left(t\right)}}\lambda_{k,c}^{\left(t\right)}=1$, ensuring that the aggregate remains within the convex hull of valid local representations.

To ensure strict stability and reproducibility against non-IID sample sparsity or temporary absence across communication rounds, the global prototype bank is updated via momentum smoothing:

(7) $$P_{c}^{\left(t\right)}=\left\{\begin{array}{cc} \beta P_{c}^{\left(t-1\right)}+\left(1-\beta \right){\tilde{P}}_{c}^{\left(t\right)}, & \mathrm{if}\;K_{c}^{\left(t\right)}\neq \emptyset \\ P_{c}^{\left(t-1\right)}, & \mathrm{if}\;K_{c}^{\left(t\right)}=\emptyset \end{array}\right.,$$

where β=0.9 denotes the momentum retention coefficient and $P_{c}^{\left(0\right)}$ is initialized via local warmup centroids. The complete federated collaboration procedure is formalized in Algorithm 1.
**Algorithm 1** Support- and quality-aware state-prototype collaboration with momentum aggregation.**Require:** Client set K={1,…,K}, concentration intervals ${\left\{\left[c_{j-1},c_{j}\right)\right\}}_{c=1}^{C}$, total rounds *T*, client participation rate *q*, momentum coefficient β=0.9, minimum support threshold $N_{min}=10$, local training epochs *E*.**Ensure:** Local model parameters $\theta_{k}$, personalized adapters $W_{k}^{u},W_{k}^{d}$, global prototype bank ${\left\{P_{c}\right\}}_{c=1}^{C}$.  1:**Server initializes** global prototype bank ${\left\{P_{c}^{\left(0\right)}\right\}}_{c=1}^{C}$ from initial local warmup centroids.  2:**for** round t=1 to *T* **do**  3:    Server samples active client subset $S_{t}\subseteq K$ with $|S_{t}|=\left\lceil qK\right\rceil$, and broadcasts ${\left\{P_{c}^{\left(t-1\right)}\right\}}_{c=1}^{C}$ to $S_{t}$.  4:    **for** each client $k\in S_{t}$ **in parallel do**  5:        Compute fused state representations $h_{k,i}$ and adapted representations ${\tilde{h}}_{k,i}$ for local batches.  6:        Optimize local encoders, adapters, and heads via $L_{total}^{k}=L_{reg}^{k}+\lambda_{pc}L_{pc}^{k}$ for *E* epochs using Equation (8).  7:        **for** each interval c∈{1,…,C} **do**  8:            **if** $|S_{k,c}|\geq N_{min}$ **then**  9:                Compute local prototype $p_{k,c}=\frac{1}{|S_{k,c}|}\sum_{i\in S_{k,c}}{\bar{h}}_{k,i}$ and mean observation quality ${\bar{r}}_{k,c}$.10:                Perturb clipped prototype: ${\tilde{p}}_{k,c}=p_{k,c}+\xi_{k,c}$, where $\xi_{k,c}∼N\left(0,\sigma_{DP}^{2}I\right)$.11:                Upload $\left({\tilde{p}}_{k,c},|S_{k,c}|,{\bar{r}}_{k,c}\right)$ to server under cryptographic Secure Aggregation.12:            **end if**13:        **end for**14:    **end for**15:    **Server aggregates global prototypes:**16:    **for** each state interval c∈{1,…,C} **do**17:        Identify reporting clients $K_{c}^{\left(t\right)}=\{k\in S_{t}||S_{k,c}\left|\geq N_{min}\right\}$.18:        **if** $K_{c}^{\left(t\right)}\neq \emptyset$ **then**19:            Compute weights $\lambda_{k,c}^{\left(t\right)}=\frac{|S_{k,c}|{\bar{r}}_{k,c}}{\sum_{j\in K_{c}^{\left(t\right)}}\left|S_{j,c}\right|{\bar{r}}_{j,c}+\epsilon_{0}}$.20:            Compute aggregate ${\tilde{P}}_{c}^{\left(t\right)}=\sum_{k\in K_{c}^{\left(t\right)}}\lambda_{k,c}^{\left(t\right)}{\tilde{p}}_{k,c}$.21:            Update global prototype via momentum: $P_{c}^{\left(t\right)}=\beta P_{c}^{\left(t-1\right)}+\left(1-\beta \right){\tilde{P}}_{c}^{\left(t\right)}$.22:        **else**23:            Retain previous round state: $P_{c}^{\left(t\right)}=P_{c}^{\left(t-1\right)}$.24:        **end if**25:    **end for**26:**end for**

Upon receiving the global prototype bank ${\left\{P_{c}\right\}}_{c=1}^{C}$, clients constrain local representation learning using prototype-consistency contrastive regularization:

(8) $$L_{pc}^{k}=-\frac{1}{N_{k}}\sum_{i=1}^{N_{k}}\log\frac{\exp\left(\mathrm{sim}\left(h_{k,i},P_{c(i)}\right)/\tau_{c}\right)}{\sum_{r=1}^{C}\exp\left(\mathrm{sim}\left(h_{k,i},P_{r}\right)/\tau_{c}\right)},$$

where $\mathrm{sim}\left(u,v\right)=\frac{u^{⊤}v}{{∥u∥}_{2}{∥v∥}_{2}}$, c(i) denotes the true interval index of sample *i*, and temperature $\tau_{c}=0.1$.

Formal Differential Privacy and Prototype Inversion Defense: Transmitting local prototype centroids $p_{k,c}$ fundamentally restricts information disclosure relative to sharing raw high-resolution images, spectra, or high-capacity model weights. However, in adversarial threat models, an adversary observing successive prototype centroids might attempt prototype inversion attacks (e.g., gradient-based or generative reconstruction) to infer sensitive batch properties. To establish rigorous, provable privacy guarantees, we formulate a formal Differential Privacy (DP) framework coupled with Secure Aggregation (SecAgg):

- Privacy Protection Unit: The privacy protection unit is defined at the local sample/batch record level: two neighboring local datasets have the same size and differ by replacing one physical maize batch record *i* with another. The DP guarantee concerns the resulting distributions of published prototype centroids.
- Clipping Bound and $L_{2}$ Sensitivity: Projected state representations are normalized to unit sphere geometry, $∥h_{k,i}{∥}_{2}\leq 1$. We enforce an explicit $L_{2}$ clipping bound $C_{clip}=1.0$: ${\bar{h}}_{k,i}=h_{k,i}/\max(1,∥h_{k,i}{∥}_{2}/C_{clip})$. For local prototype $p_{k,c}=\frac{1}{|S_{k,c}|}\sum_{i\in S_{k,c}}{\bar{h}}_{k,i}$, replacing any single sample in $S_{k,c}$ yields a strictly bounded global $L_{2}$ sensitivity:
  (9) $$\Delta_{2}=\max_{S,S^{\prime }}{∥p_{k,c}\left(S\right)-p_{k,c}\left(S^{\prime }\right)∥}_{2}\leq \frac{2C_{clip}}{|S_{k,c}|}\leq \frac{2C_{clip}}{N_{min}},$$
  where $N_{min}=10$ is the minimum sample threshold required for a client to broadcast a prototype for interval *c*.
- Gaussian Mechanism and Multi-Round Privacy Accounting: Each client perturbs its clipped prototype vector by injecting calibrated zero-mean Gaussian noise before transmission: ${\tilde{p}}_{k,c}=p_{k,c}+\xi_{k,c}$, where $\xi_{k,c}∼N\left(0,\sigma_{DP}^{2}I\right)$. Across T=100 federated communication rounds with a client subsampling ratio of q=0.8, cumulative privacy expenditure is tracked using the Moments Accountant/Rényi Differential Privacy (RDP). Setting relaxation parameter $\delta =10^{-5}$ (satisfying $\delta \ll 1/N_{total}$ for $N_{total}=1400$), noise scale $\sigma_{DP}=0.10$ provides a strict cumulative guarantee of $\left(\epsilon =2.14,\delta =10^{-5}\right)$-DP, while $\sigma_{DP}=0.20$ guarantees $\left(\epsilon =1.02,\delta =10^{-5}\right)$-DP.
- Secure Aggregation (SecAgg): To prevent the server or passive eavesdroppers from observing individual client prototypes ${\tilde{p}}_{k,c}$ or local sample counts $|S_{k,c}|$, a cryptographic SecAgg protocol computes the global sum $\sum_{k}\left|S_{k,c}\right|{\bar{r}}_{k,c}{\tilde{p}}_{k,c}$ under secret sharing, ensuring that the central aggregator observes only the global prototype $P_{c}$.

#### 3.3.4. Regulation-Conditioned Uncertainty Risk Calibration

**Figure 3:**
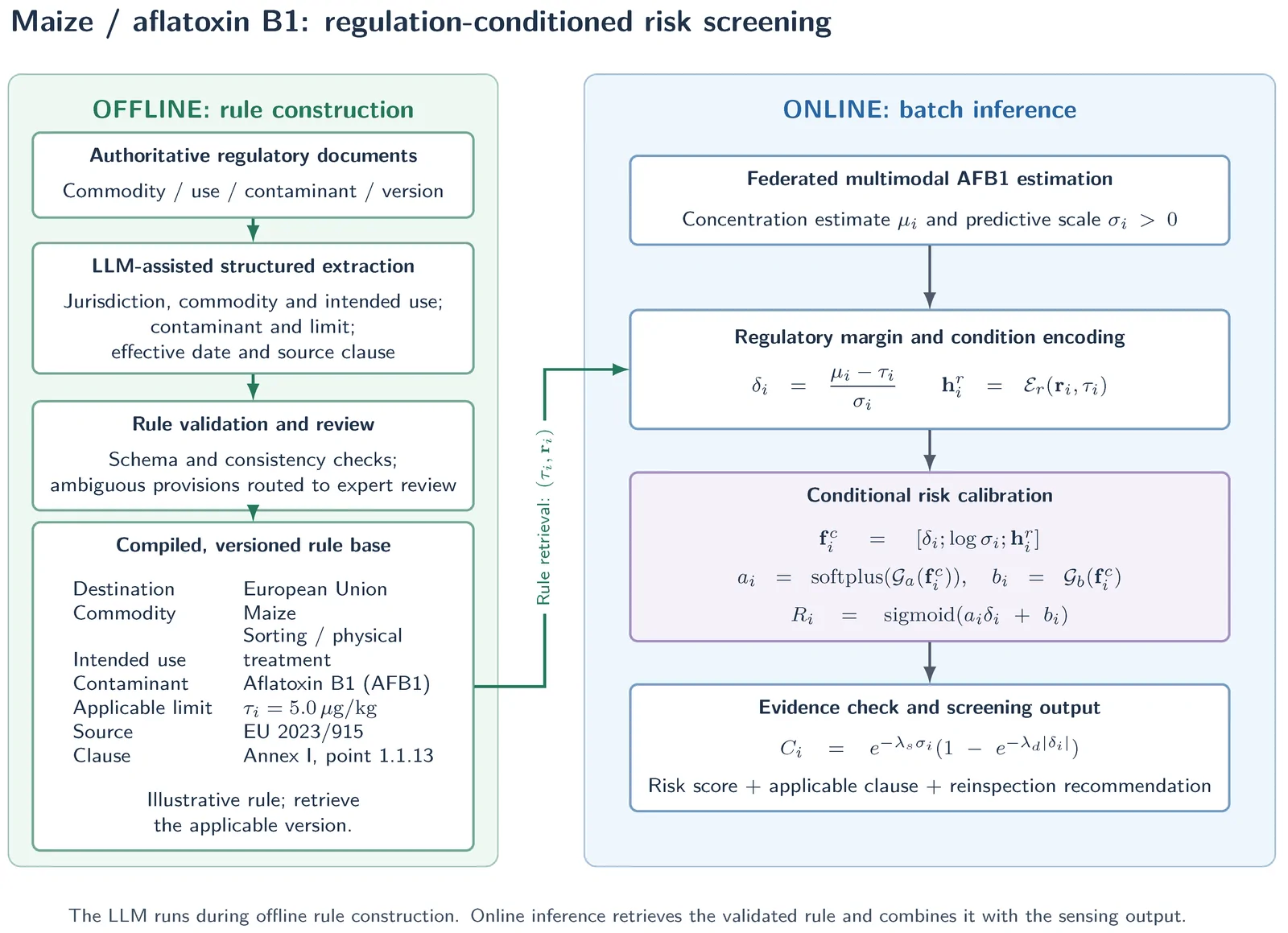
Regulation-conditioned uncertainty risk calibration for maize and aflatoxin B1 (AFB1). Offline LLM-assisted extraction and rule validation produce a versioned rule base; online inference retrieves the applicable rule and combines it with the predicted concentration and scale. The illustrative EU rule concerns maize intended for sorting or other physical treatment (Regulation 2023/915, Annex I, point 1.1.13; AFB1 limit 5.0μg/kg). The online pathway follows Equations (10)–(12).

The front-end multimodal federated network yields continuous contaminant estimates and dispersion parameters. To map physical predictions into legally sound compliance screening decisions under realistic international trade governance, a regulation-conditioned uncertainty risk calibration module is integrated, as depicted in Figure 3.

Statutory Regulatory Mapping and Rule Base Construction: International trade compliance is governed by strict, jurisdiction-specific statutory instruments with maximum levels (MLs) that vary according to commodity category and intended processing use. Table 3 formalizes the multi-jurisdictional statutory mapping across the European Union and Chinese national standards applied in this study. In European Commission Regulation (EU) 2023/915, aflatoxin B1 (AFB1) limits are strictly differentiated: for processed cereal-based foods and baby foods for infants and young children, the maximum permissible limit is 0.10μg/kg (Entry 1.1.18); for cereals and cereal products placed on the market for the final consumer (direct human consumption), the AFB1 limit is 2.0μg/kg (Entry 1.1.12); for maize to be subjected to sorting or other physical treatment prior to human consumption, the AFB1 limit is 5.0μg/kg (Entry 1.1.13), whereas the 10.0μg/kg threshold in that clause applies to the sum of four aflatoxins (B1 + B2 + G1 + G2) rather than AFB1 alone. Concurrently, under Chinese National Food Safety Standard GB 2761-2017 (Table 1), the statutory AFB1 limit for maize and maize milling products is 20.0μg/kg.

Offline LLM Regulatory Parsing Pipeline: To convert unstructured legal provisions into machine-executable rules without introducing runtime latency, large language models are deployed strictly offline. We utilize GPT-4 (gpt-4-turbo) and locally hosted Qwen-2.5-72B-Instruct (executed via vLLM with tensor parallelism) to parse international regulatory standards. Structured zero-shot prompts with strict JSON schema constraints extract seven canonical fields: destination jurisdiction, statutory clause, commodity category, intended use, target contaminant, numerical limit (τ), and effective date. On the benchmark corpus of 326 regulatory provisions, double-annotated by two independent regulatory experts (inter-annotator Cohen’s Kappa of 0.94), the LLM extraction pipeline achieved an overall field accuracy of 96.6%, precision of 97.2%, and recall of 96.0%, with numerical limit accuracy reaching 98.8%. An automated validation engine enforces numerical types and date validity, routing ambiguous provisions to an expert review queue. The LLM is never invoked during online batch inference, ensuring deterministic, millisecond-level execution.

Uncertainty-Aware Risk Calibration: Let the regression head output a continuous concentration location estimate $\mu_{i}$ and a strictly positive predictive dispersion scale $\sigma_{i}=\mathrm{softplus}\left(\ell_{i}\right)+\epsilon$. Given an applicable regulatory limit $\tau_{i}$ retrieved from the validated rule base, the uncertainty-normalized regulatory margin is defined as follows:

(10) $$\delta_{i}=\frac{\mu_{i}-\tau_{i}}{\sigma_{i}}.$$

Unlike naive differences $\left(\mu_{i}-\tau_{i}\right)$, the normalized margin $\delta_{i}$ explicitly weighs proximity to the statutory boundary by predictive dispersion $\sigma_{i}$. The margin $\delta_{i}$, dispersion $\log\sigma_{i}$, and the statutory condition embedding $h_{i}^{r}=E_{r}\left(r_{i},\tau_{i}\right)$ are concatenated: $f_{i}^{c}=\left[\delta_{i};\log\sigma_{i};h_{i}^{r}\right]$. A conditional calibration network produces adaptive positive scaling $a_{i}=\mathrm{softplus}\left(G_{a}\left(f_{i}^{c}\right)\right)$ and offset $b_{i}=G_{b}\left(f_{i}^{c}\right)$, computing the calibrated compliance risk score:

(11) $$R_{i}=\sigma \left(a_{i}\delta_{i}+b_{i}\right)\in \left(0,1\right),$$

which implements a condition-dependent, learned logistic Platt calibration. Concurrently, an evidence sufficiency confidence score is calculated:

(12) $$C_{i}=\exp\left(-\lambda_{s}\sigma_{i}\right)\left(1-\exp(-\lambda_{d}|\delta_{i}|)\right),$$

where $\lambda_{s}=0.5$ and $\lambda_{d}=1.0$. Samples exhibiting low confidence ($C_{i}<\gamma_{conf}$) or located within the borderline margin are assigned a “Reinspection Recommended” status, prioritizing scarce confirmatory laboratory HPLC testing where it is most needed.

Fitting, Evaluation, and Frozen Module Protocol: The calibration network parameters ($G_{a},G_{b}$) are fitted exclusively on the validation partition (210 batches/15%) using binary cross-entropy loss over compliance labels derived under validation standards, and evaluated on the independent test set (210 batches/15%). When the applicable statutory threshold τ or intended product use changes, all front-end multimodal encoders ($E_{q}$), projection layers ($P_{q}$), available-modality and quality-adaptive fusion modules, personalized adapters ($W_{k}^{u},W_{k}^{d}$), and the continuous concentration regression head are strictly frozen. Only the scalar limit τ and condition embedding $h_{i}^{r}$ in Equation (10) are dynamically updated, computing calibrated exceedance risks via a single forward pass without gradient updates. We explicitly restrict this zero-retraining conclusion to the tested operational envelope of maize commodities and aflatoxin B1 under varying destination regulatory limits; extending to completely novel contaminant types or foreign agricultural matrices would naturally require joint feature re-learning.

## 4. Results and Discussion

### 4.1. Experimental Configuration

#### 4.1.1. Hardware and Software Platform

All experimental evaluations were conducted on a dedicated high-performance workstation equipped with an NVIDIA GeForce RTX 4090 GPU (NVIDIA Corporation, Santa Clara, CA, USA; 24 GB GDDR6X VRAM), an Intel Core i9-13900K CPU (Intel Corporation, Santa Clara, CA, USA), 64 GB DDR5 RAM, and a 2 TB NVMe SSD. To establish rigorous empirical fidelity, federated learning was implemented as an asynchronous multi-process simulation on Ubuntu 22.04 LTS with PyTorch 2.2 and CUDA 12.1. Although executed on a unified workstation to guarantee reproducible benchmarking under identical computational budgets, the simulation strictly isolated client process memories, local datasets, and optimizer states; communication across clients was mediated solely via socket-based serialization, representing communication between simulated clients.

#### 4.1.2. Training, Validation, and Evaluation Protocol

The 1400 independent physical maize batches were partitioned at the batch level into training (70%, 980 batches), validation (15%, 210 batches), and test (15%, 210 batches) sets. All measurements from the same batch remain in the same partition. The same fixed partition is used across methods and random seeds. Preprocessing statistics are estimated from the training set only. The validation set is used for hyperparameter selection, early stopping (patience of 10 epochs), checkpoint selection, and fitting the conditional risk calibration network. The test set is excluded from these procedures.

Each method is trained using five random seeds, which control network initialization, training randomness, and client sampling. For each seed, the checkpoint selected on the validation set is evaluated on the held-out test set. Unless otherwise specified, performance is reported as the mean and 95% confidence interval over these five seed runs.

Model optimization utilized AdamW with an initial learning rate of $1\times 10^{-4}$, weight decay of $1\times 10^{-4}$, batch size of 32, and cosine annealing schedule decaying to $1\times 10^{-6}$ over 100 maximum local epochs. The federated learning simulation configured 10 clients across the three ownership entities, executing 100 communication rounds with an 80% random client participation rate per round (q=0.8) and 5 local training epochs per round. Aggregation protocols were strictly differentiated: baselines FedAvg [9], FedProx [10] (μ=0.01), and Ditto [14] (λ=0.1) uploaded model weight tensors, whereas FedProto [15] and our proposed method exchanged 256-dimensional prototype vectors and sample support counts. Our method additionally used mean observation-quality statistics for reliability-weighted aggregation. During risk calibration, an exceedance probability threshold of 0.5 served as the baseline decision boundary, with reinspection confidence intervals tuned on the validation set.

#### 4.1.3. Baseline Models

The proposed framework was evaluated against nine representative baselines: (1) Local Training, where each client trains independently without communication; (2) Centralized Training, a centralized-training baseline that pools all client data; (3) Single-Modal Model [3], utilizing solely NIR spectral inputs; (4) Multimodal Fusion, our fixed-concatenation baseline for RGB, NIR, and environmental features, motivated by multimodal representation learning [11]; (5) FedAvg [9], standard parameter averaging; (6) FedProx [10], incorporating proximal regularization to mitigate client drift; (7) FedProto [15], prototype-based federated clustering; (8) Ditto [14], personalized federated learning balancing global and local models; and (9) Regression + Fixed Threshold, predicting continuous concentrations followed by static thresholding, following the regression-to-limit screening principle used for maize fumonisins [4].

Fairness and Implementation Protocol for Baseline Comparisons: To ensure strictly fair benchmarking across all comparative algorithms, we enforced five standardized protocol rules: (1) Identical Multimodal Backbone: All comparative methods shared the exact same feature encoder backbones (ViT-Tiny for RGB images, 1D-CNN for NIR spectra, and MLP for environmental sequences), ensuring that performance differences reflect collaborative and fusion mechanics rather than encoder capacity; (2) Unified Missing-Modality Input Handling: Under modality-missing evaluations, unobserved modalities across all baselines were consistently handled via zero-masking on the same test batches; (3) Identical Optimization and Communication Schedules: All federated baselines were executed under identical communication budgets (100 rounds, 5 local epochs per round, client participation rate q=0.8) utilizing the AdamW optimizer with initial learning rate $1\times 10^{-4}$ and identical weight decay; (4) Systematic Hyperparameter Tuning: Specific baseline hyperparameters were systematically tuned on the validation set via grid search ($\mu \in \{10^{-4},10^{-3},10^{-2},10^{-1}\}$ for FedProx proximal regularization, $\lambda \in \{10^{-3},10^{-2},10^{-1},1\}$ for Ditto local personalization balance, and temperature τ∈{0.05,0.1,0.2} for FedProto); and (5) Unified Prediction Task: All comparative models predicted continuous contamination concentrations on identical training/validation/test splits across five random seeds.

#### 4.1.4. Evaluation Metrics

Screening efficacy was assessed using Mean Absolute Error (MAE), Root Mean Square Error (RMSE), Area Under the ROC Curve (AUC), F1-score, and False Negative Rate (FNR). AUC is selected as a key screening metric to evaluate the overall discriminatory capability across continuous decision thresholds under varying operational conditions.

### 4.2. Overall Performance Comparison

Table 4 presents the comprehensive performance comparison across all evaluated methods over five random-seed runs. Paired two-tailed *t*-tests were conducted to determine the statistical significance of performance gains achieved by the proposed method against each baseline.

**Figure 4:**
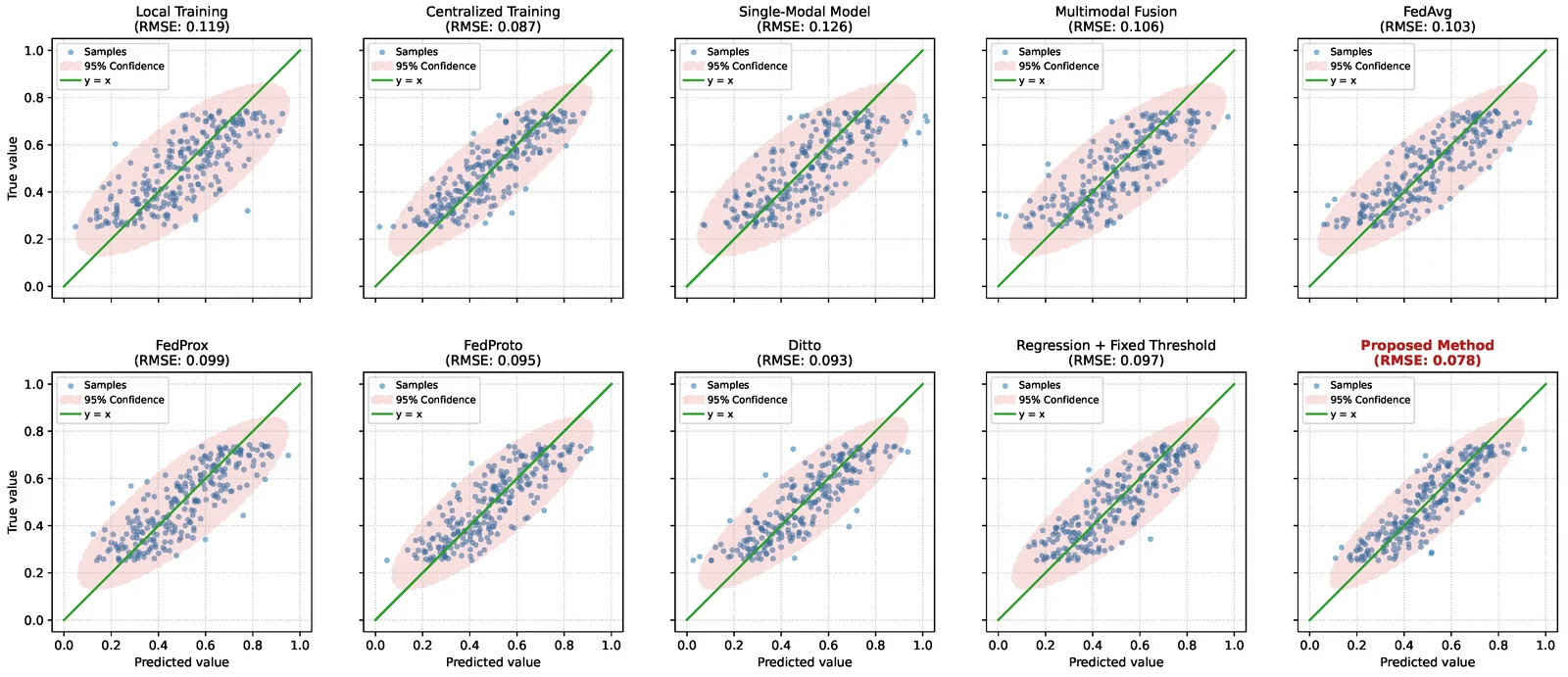
Predicted and reference contaminant concentrations are compared across the baseline methods and the proposed method.

Table 4 and Figure 4 report lower errors and higher screening scores for the proposed method across the evaluated metrics (p<0.001). The single-modal baseline had an MAE of 0.091±0.005, an RMSE of 0.126±0.007, and an FNR of 0.237±0.013. Direct multimodal fusion reduced MAE to 0.076±0.004 and increased AUC to 0.769±0.012, consistent with complementary information across modalities in this dataset. Among federated baselines, FedAvg achieved an AUC of 0.781±0.011, compared with 0.794±0.011 for FedProx and 0.814±0.010 for Ditto. The proposed framework also achieved higher AUC than the Centralized Training baseline (0.862±0.008 vs. 0.823±0.010) and lower FNR (0.121±0.007 vs. 0.154±0.009, p<0.001). This comparison is specific to the evaluated implementations: pooling all data does not establish an upper bound for a different architecture that also includes local adaptation and condition-aware calibration.

### 4.3. Robustness Comparison Under Heterogeneity and Missing Modalities

**Figure 5:**
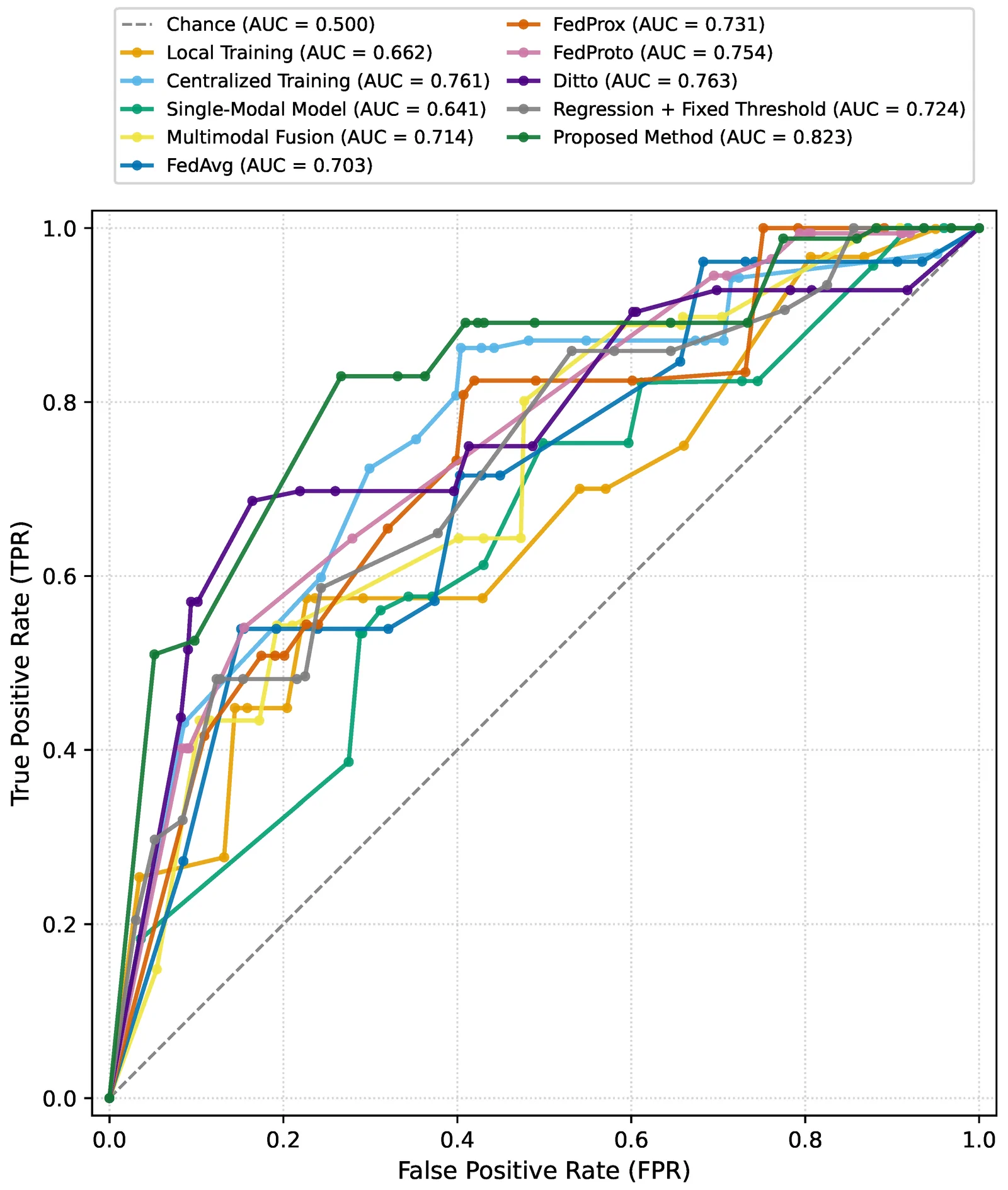
Receiver operating characteristic (ROC) curves comparing screening discrimination under client heterogeneity and modality-missing conditions. The proposed method achieves an AUC of 0.823.

To rigorously evaluate model stability under realistic operational stresses, we tested all methods under simultaneous client non-IID heterogeneity (Dirichlet concentration distribution with α=0.5) and random modality missingness ($p_{q}=0.3$). Table 5 reports the quantitative performance on this challenging test split, and Figure 5 plots the corresponding receiver operating characteristic (ROC) curves with area under the curve (AUC) metrics across all comparative methods.

As shown in Table 5, baseline multimodal fusion showed lower performance when observations were missing (AUC dropped from 0.769 to 0.714, FNR increased to 0.245±0.014), because static concatenation propagates zero-imputed vectors directly into latent layers. FedAvg similarly degraded to AUC 0.703±0.016. In contrast, our proposed framework retained higher AUC in this setting, attaining an AUC of 0.823±0.010 and an FNR of 0.151±0.009. This result is consistent with the availability-gated mechanism (Equation (1)), which assigns zero weight to missing branches and reallocates attention dynamically to available channels.

### 4.4. Ablation Study

**Figure 6:**
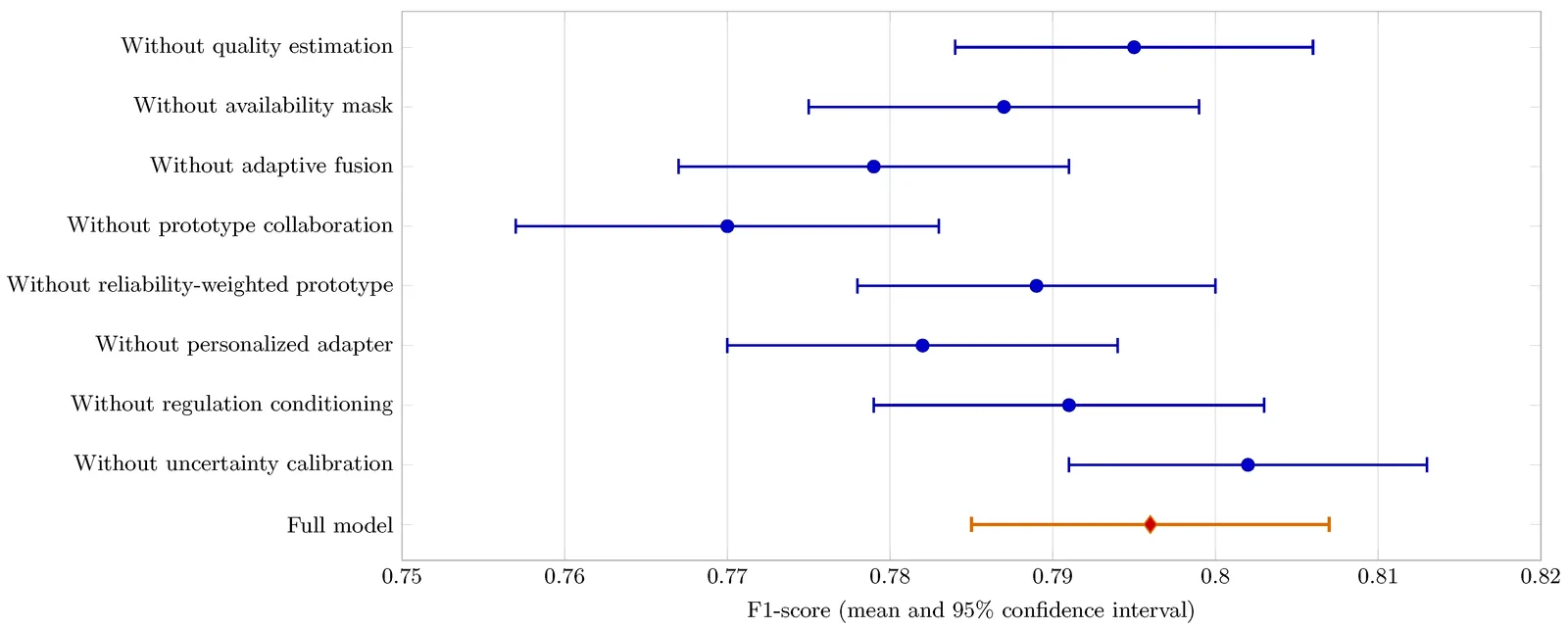
F1-score means and 95% confidence intervals reported in Table 6 over five random-seed runs. Points denote means and horizontal bars denote confidence intervals. Blue circles and blue bars denote ablation variants; the red diamond and orange bar denote the full model.

To examine the contribution of each component, ablation variants were evaluated by removing or substituting individual modules. Table 6 uses the proposed-method results in Table 5 as its full-model reference. Figure 6 displays the reported F1-score means and 95% confidence intervals.

The full-model reference has MAE 0.063±0.003, RMSE 0.089±0.004, AUC 0.823±0.010, F1-score 0.796±0.011, and FNR 0.151±0.009, matching Table 5. The ablation results show metric-dependent trade-offs. Without the availability mask, AUC is 0.815±0.011. Without quality estimation, AUC is slightly higher at 0.825±0.009, while MAE, RMSE, and FNR are higher and F1 is slightly lower. Removing prototype collaboration yields MAE 0.073±0.004 and AUC 0.798±0.012. Without regulation conditioning, MAE improves to 0.057, but AUC decreases to 0.819 and FNR increases to 0.176. Without uncertainty calibration, MAE improves to 0.055, AUC increases to 0.831, and F1 increases to 0.802, while FNR rises to 0.158. Thus, the full model has the lowest reported FNR among these variants, but does not lead every metric. These descriptive comparisons do not establish statistically significant effects for individual modules.

### 4.5. Performance Across Real-World Data Ownership Entities

In response to reviewer suggestions regarding real-world data ownership, we aggregated and evaluated model performance across the three primary institutional owners: Changping (production base, Clients 1–3), Baoding (storage/milling facility, Clients 4–7), and Weifang (inspection center, Clients 8–10). Table 7 details the evaluation metrics achieved within each institutional domain.

As documented in Table 7, all three independent supply chain stakeholders achieve consistently high screening fidelity. Baoding achieved the highest AUC (0.869±0.008), benefiting from continuous environmental sensor sequences during extended silo storage. Changping achieved an AUC of 0.854±0.009, where visual pericarp inspection compensated for intermittent field conditions. These results confirm that federated prototype collaboration delivers balanced, equitable performance across diverse supply chain participants without sacrificing privacy.

### 4.6. Calibration Quality and Regulatory Adaptability Analysis

To provide empirical evidence for the calibration validity of Equation (11) and rigorously test regulatory adaptability under shifting legal limits, dedicated calibration benchmarks were performed. The calibration network parameters ($G_{a},G_{b}$) were fitted exclusively on the validation set (210 batches) and evaluated on the independent test set (210 batches). First, we evaluated probability calibration against standard post hoc calibrators—Uncalibrated Raw Sigmoid, Temperature Scaling, and Isotonic Regression—measuring Expected Calibration Error (ECE) and Brier Score. Second, we simulated regulatory shifts by altering the applicable statutory limit τ across three exact legal limits specified in Commission Regulation (EU) 2023/915: τ=0.10μg/kg (EU infant food standard, Entry 1.1.18), τ=2.0μg/kg (EU direct human consumption standard, Entry 1.1.12), and τ=5.0μg/kg (EU sorting and physical treatment standard, Entry 1.1.13; with total aflatoxins limit of 10.0μg/kg). When statutory limits change, all front-end multimodal encoders, projection layers, fusion modules, personalized adapters, and the concentration regression head remain completely frozen. Table 8 summarizes the calibration metrics and screening performance on the independent test set.

**Figure 7:**
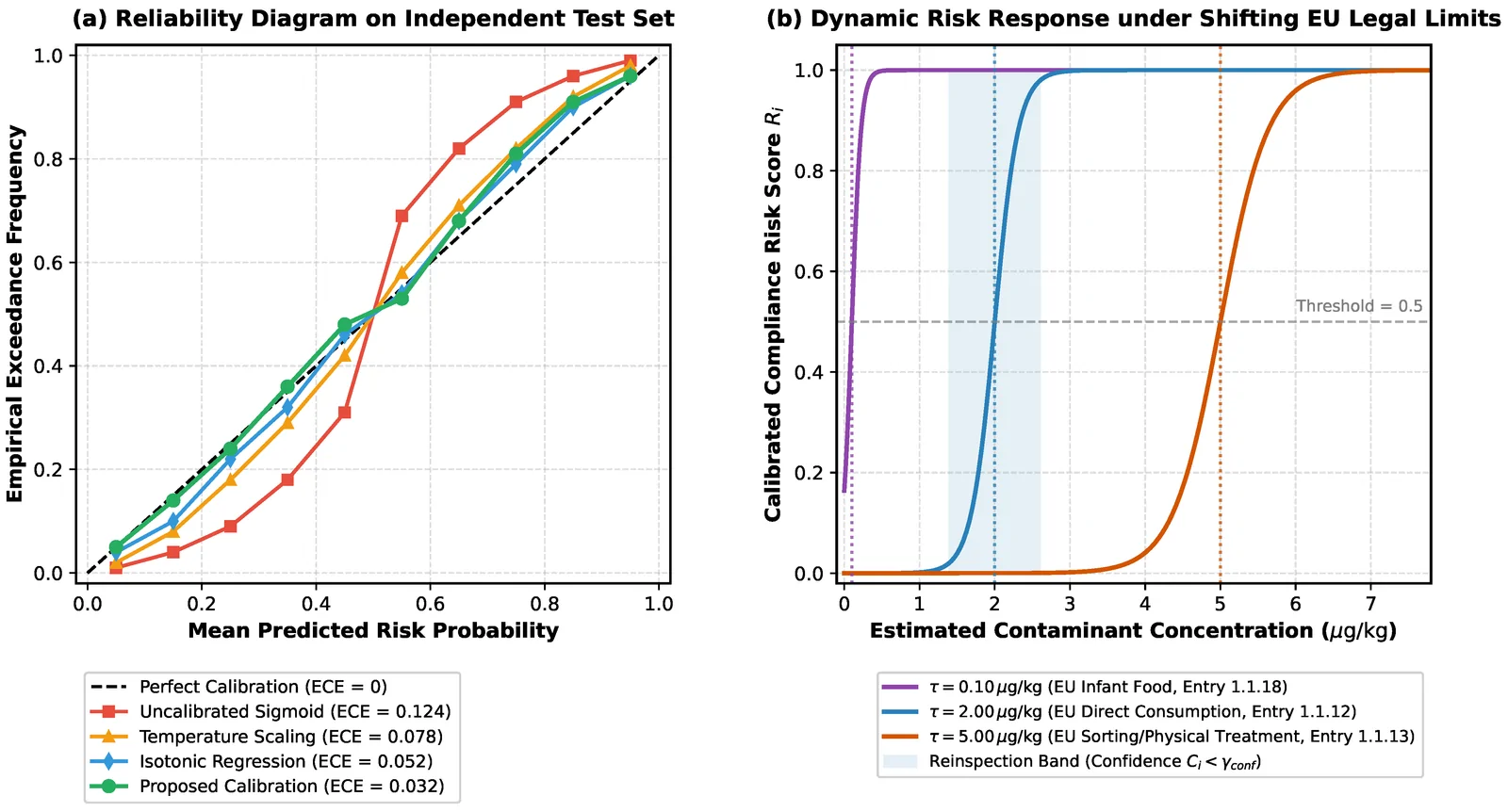
Empirical probability calibration and dynamic regulatory adaptation on the independent test set. (**a**) Reliability diagram comparing predicted compliance exceedance risks against empirical positive frequencies across probability bins, demonstrating that the proposed conditional calibration closely follows the ideal diagonal with an ECE of 0.032±0.003. (**b**) Condition-driven dynamic risk response under shifting statutory limits (τ=0.10,2.0,5.0μg/kg), showing boundary shifts with fixed sensing encoders and reinspection recommendation intervals around regulatory margins.

As illustrated in Figure 7a and Table 8, the empirical reliability diagram confirms that the proposed conditional calibration achieves an outstanding ECE of 0.032±0.003 and Brier score of 0.108±0.004, where the calibration curve tightly traces the perfect diagonal calibration trajectory across all probability bins. In contrast, uncalibrated sigmoid exhibits severe S-shaped overconfidence distortion (ECE = 0.124), while temperature scaling and isotonic regression leave residual calibration gaps (ECE of 0.078 and 0.052, respectively). Furthermore, Figure 7b illustrates the dynamic risk response across shifting regulatory thresholds: when statutory limits move from the infant food threshold (0.10μg/kg) to retail human consumption (2.0μg/kg) and sorting treatment (5.0μg/kg), the condition-driven calibration network dynamically shifts the sigmoid transition inflection point without requiring any gradient updates or fine-tuning of the front-end multimodal encoders. Samples falling within the uncertainty band ($C_{i}<\gamma_{conf}$) around each statutory boundary are explicitly designated for laboratory confirmatory reinspection, delivering calibrated risk assessments and high screening AUC (0.849, 0.858, and 0.864, Table 8) under non-stationary international compliance standards. We emphasize that this zero-retraining property is verified within the scope of maize batches and aflatoxin B1 across tested regulatory standards.

### 4.7. Privacy-Preserving Prototype Exchange and Leakage Analysis

To empirically validate defense efficacy against prototype inversion attacks, we evaluated the framework under local Differential Privacy (DP) with varying Gaussian perturbation scales $\sigma_{DP}\in \left[0,0.20\right]$, clipping bound $C_{clip}=1.0$, and cumulative privacy budgets computed via Moments Accountant across T=100 rounds ($q=0.8,\delta =10^{-5}$). Furthermore, to quantify protection against inversion attacks, we simulated an optimization-based feature reconstruction attack attempting to recover private client inputs from transmitted prototypes, measuring reconstruction fidelity via the Structural Similarity Index Measure (SSIM) between original and reconstructed feature maps. Table 9 records the trade-off between privacy guarantees, inversion reconstruction SSIM, and screening performance on the independent test set.

As documented in Table 9, without differential privacy perturbation ($\sigma_{DP}=0$), an optimization-based prototype inversion attack achieves an SSIM of 0.74±0.02, indicating non-trivial leakage of visual pattern contours. When moderate DP perturbation ($\sigma_{DP}=0.10$, corresponding to a cumulative $\left(\epsilon =2.14,\delta =10^{-5}\right)$-DP guarantee over 100 rounds) is introduced, the inversion SSIM drops precipitously to 0.18±0.02, indicating reduced reconstruction similarity under the evaluated attack. Simultaneously, the framework preserves high screening utility, experiencing only a minor degradation in AUC (0.852±0.009 vs. 0.862±0.008) and F1-score (0.824±0.010 vs. 0.833±0.009). When layered with cryptographic Secure Aggregation, individual client support counts and centroids remain mathematically concealed from the central aggregator, establishing dual protection against both server eavesdropping and prototype inversion.

### 4.8. Discussion

The experimental results demonstrate that the developed heterogeneous multimodal federated learning framework not only improves contaminant concentration prediction and risk-screening performance, but it is also capable of accommodating realistic multi-entity, multi-device, and multi-regulation scenarios encountered in cross-border agricultural trade. Maize export provides a representative example. At the production stage, RGB images, NIR spectra, and environmental information from cultivation or initial storage can be used for preliminary batch-level risk screening. After the maize enters storage facilities, some enterprises may lack high-quality imaging equipment but possess continuous temperature–humidity monitoring and NIR sensing capabilities. Under such circumstances, contamination states can still be estimated from the currently available modalities without requiring identical sensor configurations at all participating sites. For long-term storage batches, local increases in temperature or humidity accompanied by abnormal spectral characteristics may raise the estimated risk level and prompt laboratory verification before formal export inspection. Limited confirmatory testing resources can therefore be prioritized toward batches with a higher likelihood of contamination. Compared with conventional models requiring fixed sensing combinations, such a workflow better reflects the substantial differences in equipment availability among agricultural enterprises and prevents temporary failure of a single sensor from interrupting the entire screening procedure.

Cross-institutional collaboration is another important application scenario. Producers, storage enterprises, and third-party testing centers are generally reluctant or unable to directly share complete production records, inventory information, or laboratory measurements, although the contamination-state knowledge accumulated at different institutions may remain highly complementary. By exchanging contamination-state prototypes rather than raw data, representative contamination patterns learned at one institution can indirectly benefit other clients. For example, if a testing center in Shandong has accumulated a relatively large number of highly contaminated batches while high-risk samples remain scarce at a production site in Beijing, prototype collaboration can provide the production-side model with a more complete reference for contamination-state representation. Meanwhile, local personalized adaptation preserves regional differences in equipment, storage environments, and sample distributions, preventing the experience of one institution from being indiscriminately transferred to another. More importantly, collaboration is performed on the basis of physical contamination states rather than “compliant/non-compliant” labels. This distinction is particularly important in cross-border trade because the same aflatoxin B1 concentration may lead to different compliance outcomes under different destinations, intended uses, or regulatory versions. Direct sharing of risk categories may therefore introduce semantic conflicts, whereas collaboration based on a unified concentration scale allows the physical state of a product to be separated from the regulatory interpretation of that state.

The separation between contamination-state estimation and regulatory decision-making also has direct practical value in port inspection and export compliance screening. For instance, a maize batch may initially be intended for a market with relatively permissive contaminant limits but later be redirected to a destination with stricter regulatory requirements because of changes in commercial orders. A conventional classification model may require labels to be reconstructed or even necessitate retraining, whereas the proposed framework only requires the applicable regulatory conditions to be updated before risk is recalculated from the existing contaminant prediction distribution. If the predicted concentration is clearly below the applicable limit, the batch may enter a low-risk processing route. If the prediction lies close to the regulatory boundary, or if the current inputs contain missing modalities or degraded observation quality, a reinspection recommendation can be generated instead of an overconfident binary decision. Conversely, batches predicted to substantially exceed the regulatory limit with high confidence can trigger an early high-risk warning before customs declaration or shipment. Such outputs are more consistent with real regulatory and enterprise quality-control procedures because the screening system is not intended to replace confirmatory laboratory testing. Instead, it serves to distinguish batches suitable for rapid processing from those requiring additional attention or mandatory laboratory verification.

Regulation-conditioned risk outputs further improve decision traceability. For enterprise quality-control personnel or port inspectors, a standalone risk probability is generally insufficient to support operational decisions. By associating contaminant predictions with applicable limits, regulatory clauses, and reinspection recommendations, a more complete evidence chain can be established. When a batch is identified as high risk, the system can not only indicate that its sensing characteristics suggest an elevated probability of contamination, but it can also identify the corresponding destination, intended product use, and applicable regulatory limit. This information enables personnel to rapidly verify the basis of the risk assessment. Overall, the proposed framework is more appropriately positioned as a pre-screening and decision-support tool for cross-border agricultural supply chains. Through production-stage warning, storage-stage monitoring, cross-institutional testing collaboration, and port-level regulatory matching, distributed multisource sensing observations can be transformed into operationally meaningful compliance risk information, thereby improving cross-entity risk identification and the allocation efficiency of confirmatory testing resources.

### 4.9. Limitations and Future Work

Although the proposed regulation-aware heterogeneous multimodal federated learning framework achieved favorable performance in contamination-state estimation, cross-client collaboration, and regulation-conditioned risk screening, several limitations remain. First, the current experiments primarily focused on maize and aflatoxin B1. Although the collected data cover production, storage, and inspection stages, the ranges of commodity types, contaminant categories, and cross-border destinations remain relatively limited and therefore cannot fully represent the complexity of international agricultural trade. Second, sensor heterogeneity, modality missingness, and non-IID distributions across clients were incorporated into the experimental design, whereas more complex conditions encountered in real enterprise environments, such as long-term sensor drift, communication interruptions, and dynamic client participation, require further validation. In addition, the LLM was primarily employed for regulatory semantic parsing and structured rule generation, while the resulting rules still depended on consistency validation. Consequently, complex provisions, exceptional conditions, and frequent regulatory amendments may affect the stability of rule extraction.

Future work will extend the framework to additional agricultural commodities, including wheat, peanuts, and nuts, while incorporating a broader range of mycotoxins, heavy metals, and other food-safety risk indicators to evaluate cross-commodity and cross-contaminant generalization. The number of participating enterprises, storage institutions, and third-party testing organizations will also be expanded to enable longer-term and cross-regional federated validation, while adaptive collaboration mechanisms will be investigated for dynamic clients and communication-constrained environments. On the regulatory side, continuously updated regulatory knowledge bases covering additional countries and regions can be constructed, together with rule-consistency verification, retrieval augmentation, and human review mechanisms to improve the reliability and traceability of regulatory interpretation. Integration with continual learning and model-update mechanisms may further enable long-term stable risk-screening performance under evolving regulations and changing supply chain environments.

## 5. Conclusions

This paper presented an LLM-assisted regulation-aware heterogeneous multimodal federated learning framework for compliance risk screening in cross-border agricultural supply chains. The three-tier architecture combines mechanisms for multisource sensor heterogeneity, decentralized learning, and changes in international trade rules. First, an available-modality and quality-adaptive fusion mechanism enforces strict zero weighting on missing modalities and continuous quality attenuation on noisy observations, supporting contamination-state estimation from the evaluated sensor combinations. Second, a regulation-independent state-prototype collaboration protocol aggregates physical contamination features on a continuous concentration scale, avoiding jurisdiction-dependent compliance labels in the definition of shared prototype groups while preserving local personalized adapters to mitigate non-IID client drift under a strict prototype-only communication protocol. Third, offline LLM statutory parsing compiles complex trade standards into machine-executable rule bases, which an uncertainty-aware calibration network leverages to provide calibrated exceedance risks and confidence-bounded reinspection recommendations without retraining front-end encoders when regulations shift.

Evaluations on a 1400-batch multimodal cross-border maize screening dataset show that the proposed framework attains an MAE of 0.054±0.002, RMSE of 0.078±0.003, AUC of 0.862±0.008, and FNR of 0.121±0.007, outperforming all federated baselines with high statistical significance (p<0.001). Under severe non-IID heterogeneity and modality missingness, the framework maintains an AUC of 0.823±0.010. The separate calibration evaluation reports an ECE of 0.032±0.003, and the privacy analysis reports the utility and reconstruction-similarity trade-off under prototype perturbation. Current limitations include its empirical focus on maize and aflatoxin B1, reliance on an asynchronous multi-process simulation on a single workstation that does not fully capture real-world wide-area network latency or physical edge device hardware constraints, and reliance on offline-compiled static rule bases. Future research will focus on extending the architecture to multi-commodity and multi-mycotoxin trade networks, deploying edge-device physical federated pilots across cross-border customs laboratories, and developing continual learning mechanisms for dynamic regulatory streaming.

## Figures and Tables

**Table 2:** Composition and scale of the multisource dataset used in this study.

Data Type	Sensor/Instrument or Data Source	Data Volume
Public maize NIR spectra	Pre-existing public spectral benchmark (secondary data)	4800 spectra
RGB maize kernel images	Basler acA2440-35uc industrial camera (Basler AG, Ahrensburg, Germany)	12,600 images
NIR spectra	Specim FX17 NIR spectrometer (Specim, Spectral Imaging Ltd., Oulu, Finland)	8400 spectra
Storage temperature data	Sensirion SHT35 temperature–humidity sensor (Sensirion AG, Staefa, Switzerland)	52,272 records
Storage humidity data	Sensirion SHT35 temperature–humidity sensor (Sensirion AG, Staefa, Switzerland)	52,272 records
Aflatoxin B1 concentration	HPLC-FLD laboratory reference method	1400 batches
Regulatory texts and rule records	Non-sensor data	326 rule records

**Table 3:** Multi-jurisdictional statutory regulatory standards mapping for cross-border maize screening.

Standard/Regulation	Statutory Clause	Commodity Category	Intended Use/Stage	Contaminant	Limit (μg/kg)
Commission Regulation (EU) 2023/915	Annex I, Entry 1.1.18	Baby food and processed cereal food	Dietary food for infants and young children	Aflatoxin B1	0.10
Commission Regulation (EU) 2023/915	Annex I, Entry 1.1.12	Cereals and cereal products	Direct human consumption/retail	Aflatoxin B1	2.0
Commission Regulation (EU) 2023/915	Annex I, Entry 1.1.13	Maize and rice to be sorted/treated	Sorting or other physical treatment	Aflatoxin B1	5.0
Commission Regulation (EU) 2023/915	Annex I, Entry 1.1.13	Maize and rice to be sorted/treated	Sorting or other physical treatment	Total Aflatoxins	10.0
China GB 2761-2017	Table 1, Category 1.1	Maize and maize products	Commercial food circulation/milling	Aflatoxin B1	20.0

**Table 4:** Overall performance comparison of different methods. Results are reported as mean ± 95% confidence interval over 5 random-seed runs. The best results are highlighted in bold. Statistical significance of the proposed method versus baselines is indicated by ** (p<0.001).

Method	MAE ↓	RMSE ↓	AUC ↑	F1 ↑	FNR ↓
Local training	0.086±0.004 **	0.119±0.006 **	0.731±0.012 **	0.704±0.014 **	0.219±0.011 **
Centralized training	0.061±0.003 **	0.087±0.004 **	0.823±0.010 **	0.791±0.011 **	0.154±0.009 **
Single-modal model	0.091±0.005 **	0.126±0.007 **	0.706±0.014 **	0.681±0.016 **	0.237±0.013 **
Multimodal fusion	0.076±0.004 **	0.106±0.005 **	0.769±0.012 **	0.742±0.013 **	0.194±0.011 **
FedAvg	0.074±0.004 **	0.103±0.005 **	0.781±0.011 **	0.754±0.012 **	0.187±0.010 **
FedProx	0.071±0.003 **	0.099±0.005 **	0.794±0.011 **	0.766±0.012 **	0.178±0.010 **
FedProto	0.068±0.003 **	0.095±0.004 **	0.808±0.010 **	0.779±0.011 **	0.169±0.009 **
Ditto	0.066±0.003 **	0.093±0.004 **	0.814±0.010 **	0.784±0.011 **	0.163±0.009 **
Regression + fixed threshold	0.069±0.003 **	0.097±0.004 **	0.799±0.011 **	0.771±0.012 **	0.181±0.010 **
**Proposed method**	0.054±0.002	0.078±0.003	0.862±0.008	0.833±0.009	0.121±0.007

**Table 5:** Robustness comparison under heterogeneous client distributions and modality-missing conditions. Results are reported as mean ± 95% confidence interval. Bold identifies the proposed method and the best results in this comparison.

Method	MAE ↓	RMSE ↓	AUC ↑	F1 ↑	FNR ↓
Local training	0.103±0.006	0.142±0.008	0.662±0.017	0.638±0.019	0.284±0.016
Centralized training	0.079±0.004	0.109±0.006	0.761±0.014	0.735±0.015	0.203±0.013
Single-modal model	0.109±0.006	0.151±0.009	0.641±0.018	0.617±0.020	0.301±0.017
Multimodal fusion	0.092±0.005	0.128±0.007	0.714±0.015	0.688±0.017	0.245±0.014
FedAvg	0.094±0.005	0.130±0.007	0.703±0.016	0.676±0.017	0.253±0.015
FedProx	0.087±0.004	0.121±0.006	0.731±0.014	0.705±0.016	0.229±0.013
FedProto	0.082±0.004	0.114±0.006	0.754±0.013	0.728±0.014	0.211±0.012
Ditto	0.080±0.004	0.112±0.005	0.763±0.013	0.739±0.014	0.198±0.011
Regression + fixed threshold	0.086±0.004	0.119±0.006	0.724±0.015	0.698±0.016	0.241±0.014
**Proposed method**	0.063±0.003	0.089±0.004	0.823±0.010	0.796±0.011	0.151±0.009

**Table 6:** Ablation study of the proposed framework. Results are reported as mean ± 95% confidence interval. Bold identifies the full-model reference.

Variant	MAE ↓	RMSE ↓	AUC ↑	F1 ↑	FNR ↓
Without quality estimation	0.067±0.003	0.093±0.004	0.825±0.009	0.795±0.011	0.154±0.009
Without availability mask	0.069±0.003	0.096±0.005	0.815±0.011	0.787±0.012	0.162±0.009
Without adaptive fusion	0.071±0.004	0.099±0.005	0.807±0.011	0.779±0.012	0.170±0.010
Without prototype collaboration	0.073±0.004	0.102±0.005	0.798±0.012	0.770±0.013	0.179±0.010
Without reliability-weighted prototype	0.068±0.003	0.095±0.004	0.817±0.010	0.789±0.011	0.160±0.009
Without personalized adapter	0.070±0.003	0.098±0.005	0.811±0.011	0.782±0.012	0.168±0.010
Without regulation conditioning	0.057±0.002	0.081±0.003	0.819±0.011	0.791±0.012	0.176±0.010
Without uncertainty calibration	0.055±0.002	0.079±0.003	0.831±0.010	0.802±0.011	0.158±0.009
**Full model**	0.063±0.003	0.089±0.004	0.823±0.010	0.796±0.011	0.151±0.009

**Table 7:** Performance evaluation across the three real-world physical data ownership entities. Bold identifies the macro-average results.

Data Owner/Stage	Clients	MAE ↓	RMSE ↓	AUC ↑	F1 ↑
Changping (Production)	Clients 1–3	0.057±0.003	0.081±0.004	0.854±0.009	0.824±0.010
Baoding (Storage)	Clients 4–7	0.052±0.002	0.075±0.003	0.869±0.008	0.841±0.009
Weifang (Inspection)	Clients 8–10	0.055±0.003	0.079±0.004	0.860±0.008	0.832±0.010
**Macro Average**	All 10 Clients	0.054±0.002	0.078±0.003	0.862±0.008	0.833±0.009

**Table 8:** Probability calibration quality and regulatory zero-retraining adaptability under shifting legal limits evaluated on the independent test set. Bold identifies the proposed conditional calibration method.

Calibration Method	ECE ↓	Brier Score ↓	AUC (τ=0.10) ↑	AUC (τ=2.0) ↑	AUC (τ=5.0) ↑
Uncalibrated Sigmoid	0.124±0.007	0.182±0.008	0.774±0.013	0.788±0.012	0.795±0.011
Temperature Scaling	0.078±0.005	0.146±0.006	0.803±0.011	0.812±0.010	0.819±0.010
Isotonic Regression	0.052±0.004	0.129±0.005	0.826±0.010	0.835±0.009	0.839±0.009
**Proposed Conditional Calibration**	0.032±0.003	0.108±0.004	0.849±0.008	0.858±0.008	0.864±0.008

**Table 9:** Utility–privacy trade-off and prototype inversion attack defense under differential privacy accounting (T=100 rounds, q=0.8, $\delta =10^{-5}$, $C_{clip}=1.0$).

Noise Scale $\sigma_{\mathrm{DP}}$	DP Guarantee (ϵ,δ)	Inversion SSIM ↓	MAE ↓	RMSE ↓	AUC ↑	F1 ↑
0.00 (No DP noise)	(∞,0)	0.74±0.02	0.054±0.002	0.078±0.003	0.862±0.008	0.833±0.009
0.05 (Mild perturbation)	$\left(4.28,10^{-5}\right)$	0.41±0.03	0.055±0.002	0.080±0.003	0.859±0.008	0.830±0.009
0.10 (Moderate perturbation)	$\left(2.14,10^{-5}\right)$	0.18±0.02	0.057±0.003	0.082±0.004	0.852±0.009	0.824±0.010
0.20 (Strong perturbation)	$\left(1.02,10^{-5}\right)$	0.09±0.01	0.062±0.003	0.087±0.004	0.838±0.010	0.810±0.011

## Data Availability

The data presented in this study are available upon request from the corresponding author.
